# Supplementary material for: H3.3K27M-induced chromatin changes drive ectopic replication through misregulation of the JNK pathway in C. elegans
Source: Nat Commun. 2019 Jun 7;10:2529. doi: 10.1038/s41467-019-10404-9 (PMC6555832; doi:10.1038/s41467-019-10404-9)
Supplement: Supplementary file 1 — Supplementary Information [file 41467_2019_10404_MOESM1_ESM.pdf]

Supplementary Information

H3.3K27M-induced chromatin changes drive ectopic replication through misregulation of the JNK pathway in *C. elegans*

Delaney, et al.

This PDF file includes:
Supplementary Figs 1-11
Supplementary Tables 1-2

Other supplementary information not included in this file:
Supplementary Data 1 (separate .xlsx file)

Supplementary Fig. 1

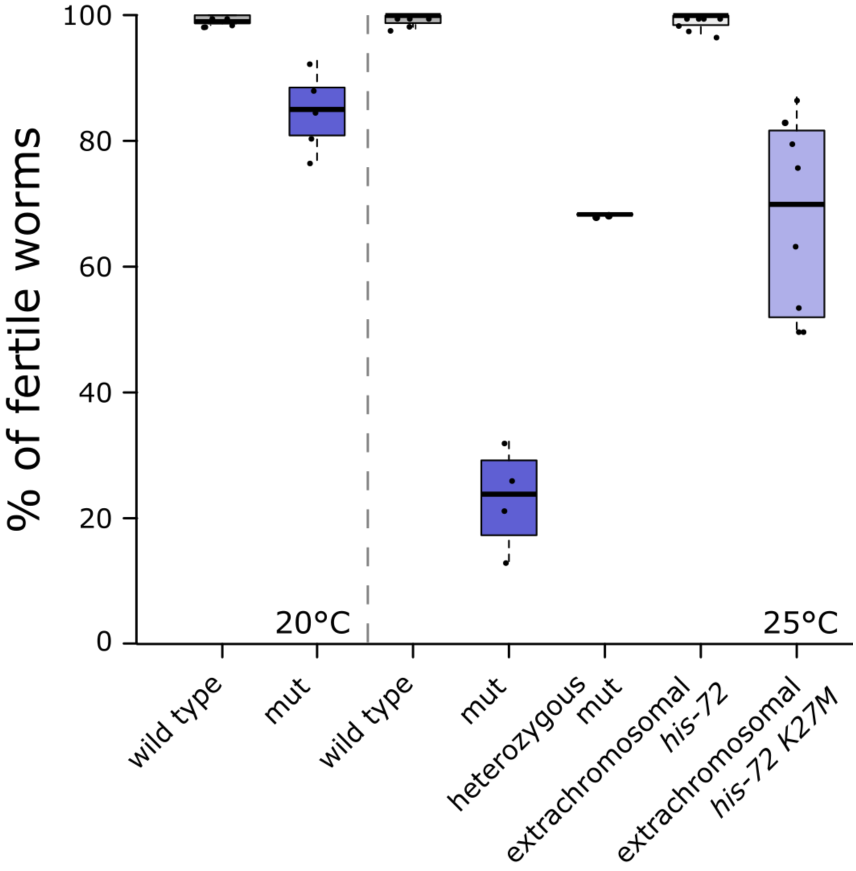

Supplementary Fig. 1. H3.3K27M mutation leads to worms sterility. Box plots illustrating levels of fertility of wild type and H3K27M mutant (mut) worms at 20°C, and of wild type, mut homozygous, mut heterozygous, extrachromosomal wild type H3.3 and extrachromosomal H3.3K27M worms at 25°C. The data points for wild type and mut homozygous at 25°C are reproduced from Fig. 1A. N=3 for all conditions except mut homozygous at 25°C (N=4) and H3.3K27M heterozygote (N=2). Source data are provided as a Source Data Supplementary Fig. 1 file.

Supplementary Fig. 2

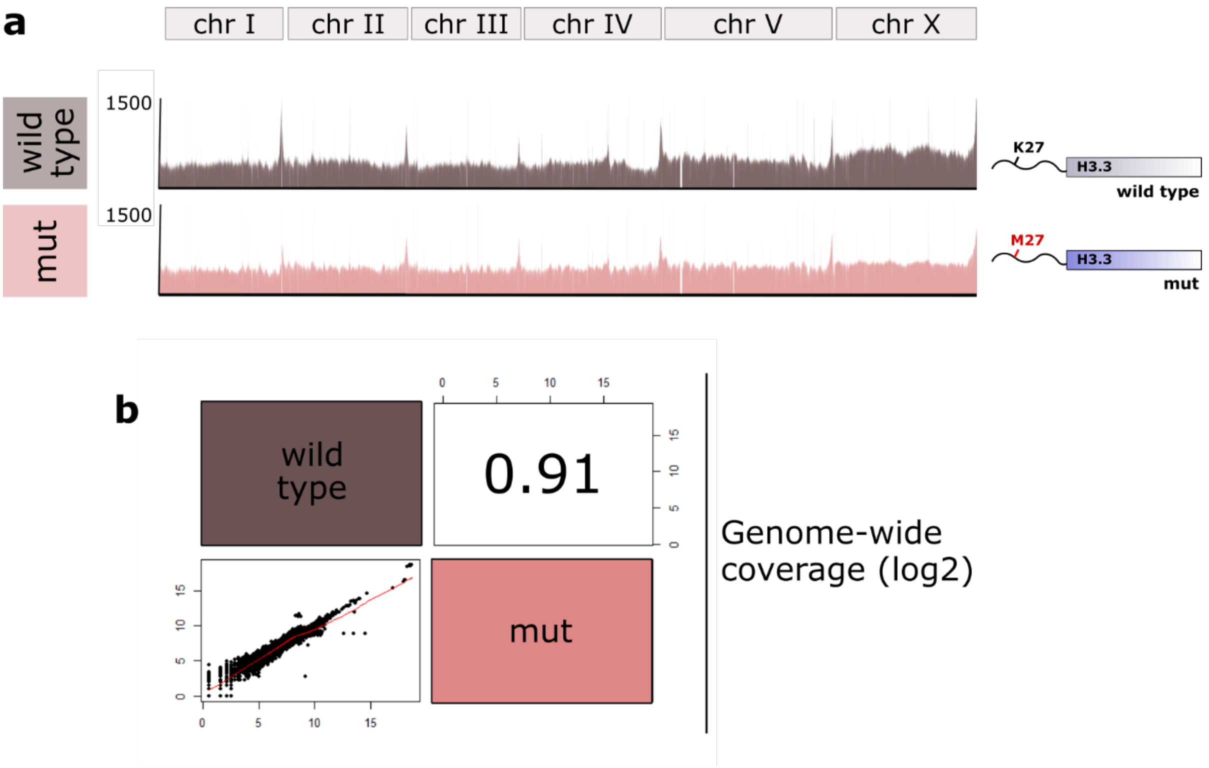

Supplementary Fig. 2. The whole genome is replicated in endomitotic oocytes. (**a**) Genome browser view of whole genome sequencing of wild type (upper track, dark pink) and endomitotic oocytes (lower track, light pink). Tracks were normalized to the total number of reads. (**b**) Genome-wide comparison between 1 kb bin coverage in wild type and endomitotic oocytes. Panel shows scatter plot and Pearson correlation.

Supplementary Fig. 3

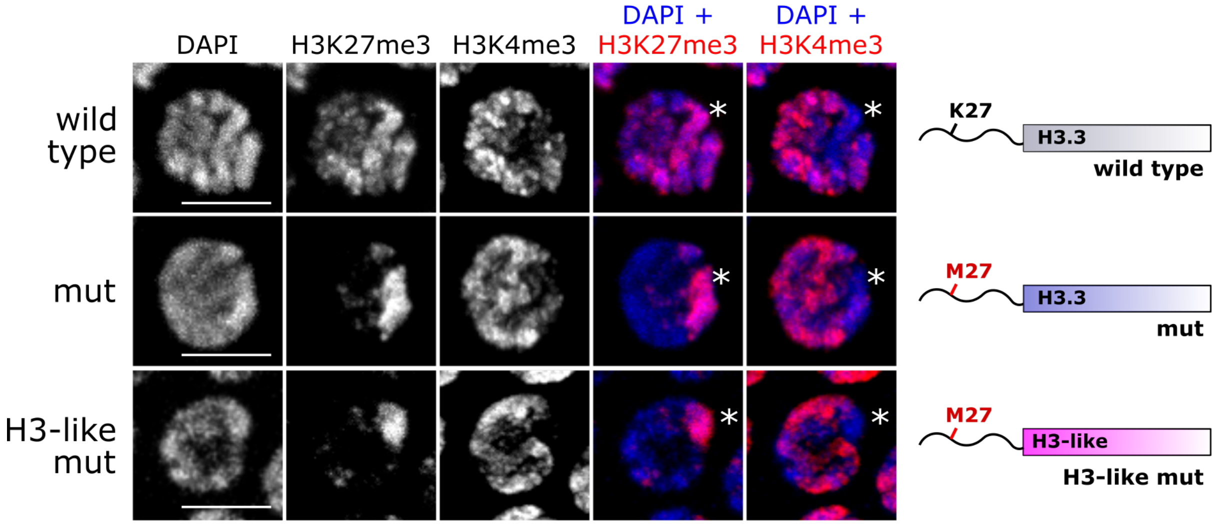

Supplementary Fig. 3. Chromosome X retains high levels of H3K27me3 in the H3.3K27M and H3-like K27M mutant cells. H3K4me3 and H3K27me3 staining of wild type, H3.3K7M mutant (mut) and H3-like K27M mutant (H3-like mut) pachytene cells are shown. Chromosome X is identified by depletion of H3K4me3, which coincides with high H3K27me3 levels in wild type and both mut and H3-like mut pachytene cells (marked by the asterisks in the merges). Scale bars represent 5 µm.

Supplementary Fig. 4

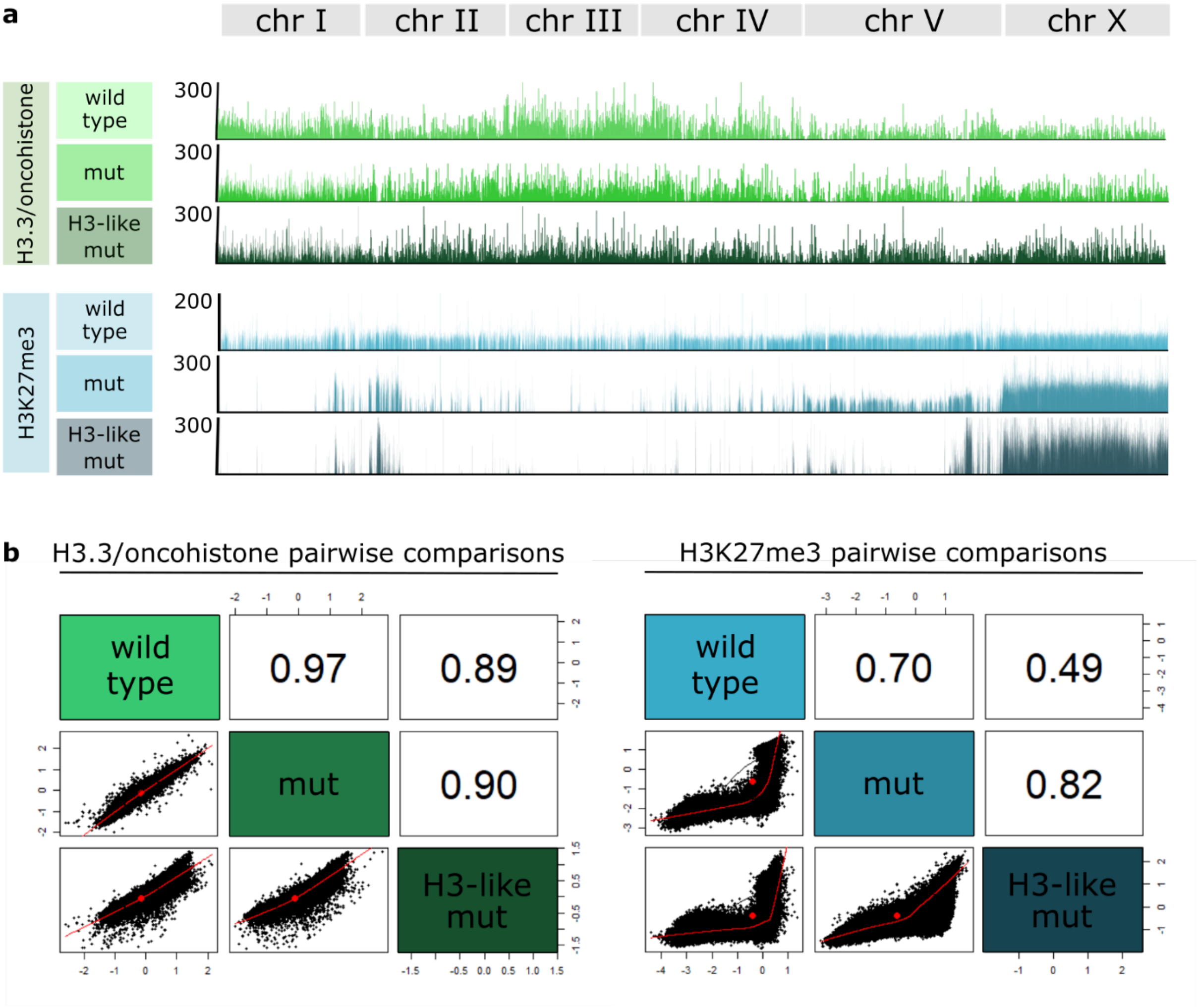

Supplementary Fig. 4. Genome wide distributions of wild type and mutant versions of H3.3 and the resulting H3K27me3 patterns. (**a**) Genome browser view of whole genome showing H3.3/oncohistone incorporation (green) and H3K27me3 distribution (blue) in wild type (light), H3.3K27M mutant (mut, medium) and H3-like K27M mutant (H3-like mut, dark) worms. (**b**) Genome-wide pairwise comparisons of H3K27me3 (left panel) and H3.3/oncohistone (right panel) levels in 1kb bins between wild type, mut and H3-like mut worms. Panels show scatter plots and Pearson correlations for each sample pair.

Supplementary Fig. 5

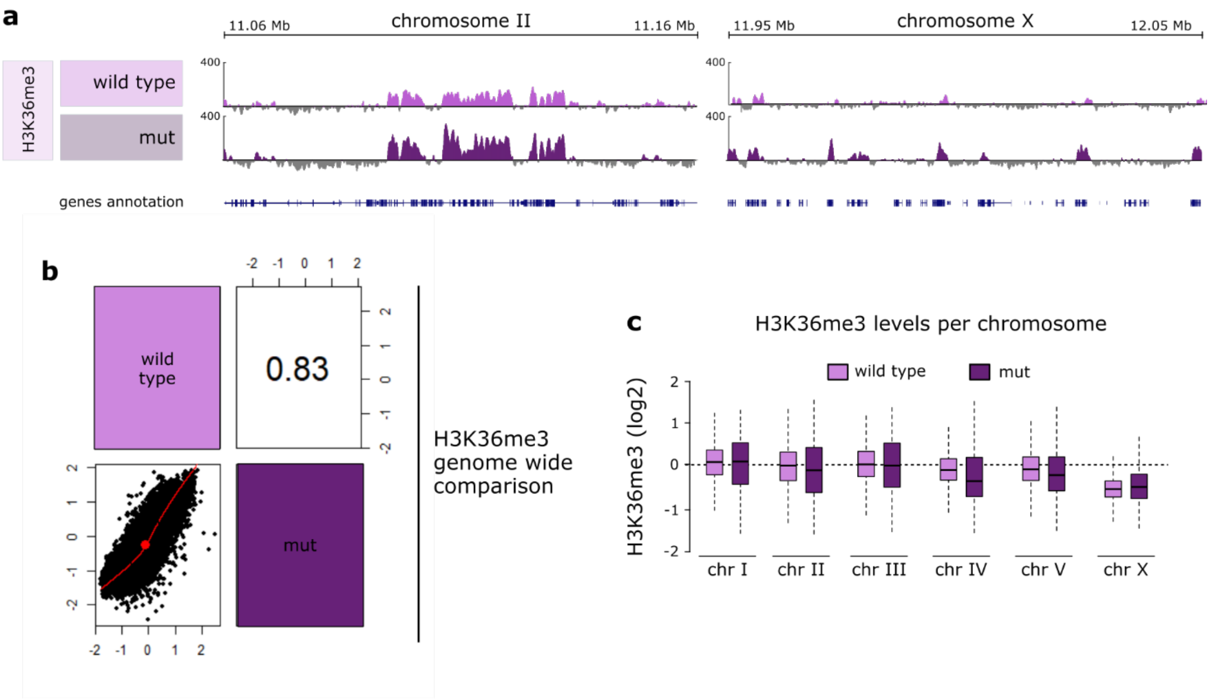

Supplementary Fig. 5. H3K36me3 distribution is not affected by the H3.3K27M mutation. (**a**) Genome browser view of 100 kb on chromosome II and X showing H3K36me3 distribution in wild type (light purple) and H3.3K7M mutant (mut, dark purple) worms. (**b**) Genome-wide comparison between wild type and mut H3K36me3 levels in 1kb bins. Panel shows scatter plot and Pearson correlation. (**c**) Box plots showing H3K36me3 occupancy per chromosome in wild type (light purple) and mut (dark purple).

Supplementary Fig. 6

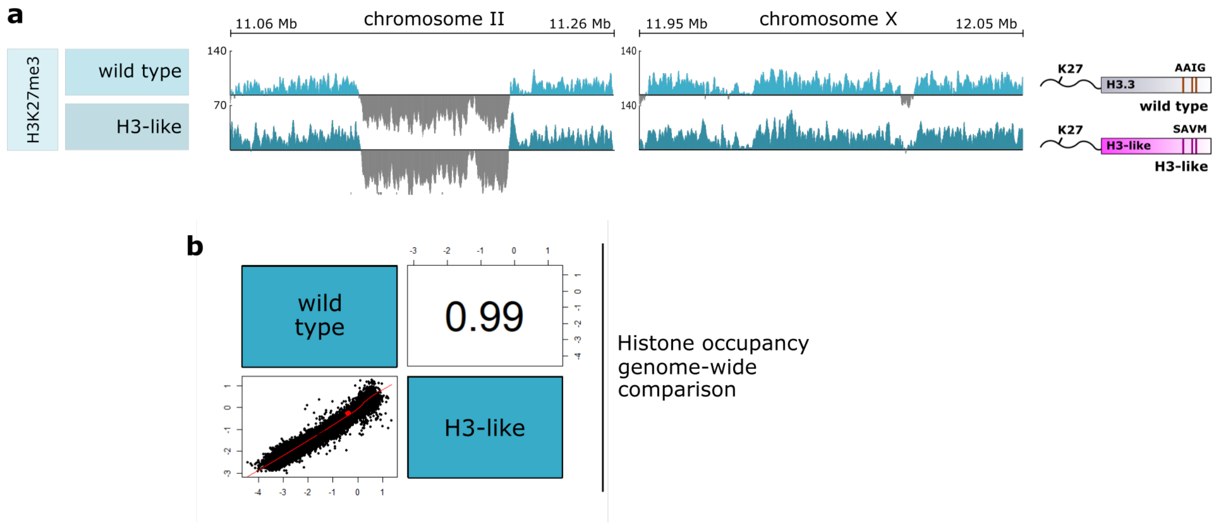

Supplementary Fig. 6. Changing the H3.3 chaperone specificity alone does not affect the H3.3K27me3 distribution. (**a**) Genome browser view of 100 kb on chromosome II and X showing H3K27me3 distribution in wild type (light blue) and H3-like (dark blue) worms (**b**) Genome-wide comparison between wild type and H3-like H3K27me3 levels in 1kb bins. Panel shows scatter plot and Pearson correlation. In both (**a**) and (**b**), the wild type data are the same dataset as shown in Fig. 3 for the wild type analysis.

Supplementary Fig. 7

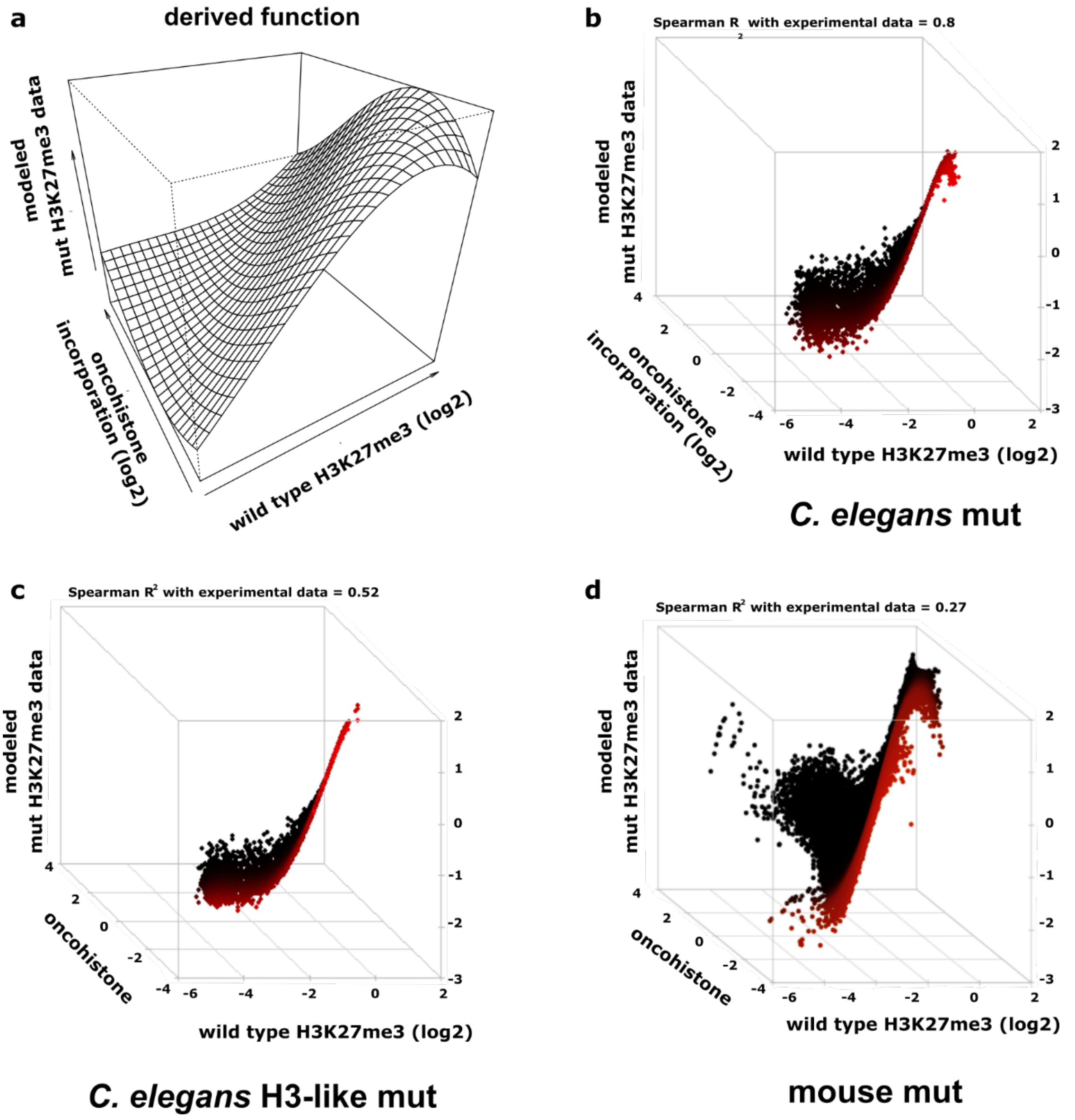

Supplementary Fig. 7. Mathematical model of mutant H3K27me3 levels as a function of oncohistone incorporation and pre-existing H3K27me3 levels. The model was derived from ChIP data of H3K27me3 in wild type and of H3K27me3 and oncohistone in H3.3K27M mutant (mut) worms. The model was then applied to predict H3K27me3 levels in the datasets from H3K27M mutant (mut) and H3-like H3K27M mutant (H3-like mut) worms as well as mouse ESCs carrying the H3.3K27M mutation using ChIP data of H3K27me3 in wild type and of the oncohistone in the corresponding mutant. (**a**) Surface representation of the function

that best describes the dependencies between wild type H3K27me3, oncohistone levels and mutant H3K27me3. (**b**) 3D representation of the model applied to the data from mut worms, from which the model was derived. (**c**) 3D representation of the model applied to the data from H3-like mut worms. (**d**) 3D representation of the model applied to the data from mouse H3.3K27M mutant ESCs (mut ESCs). Data points in are color coded based on H3.3K27M incorporation values from high (black) to low (red).

Supplementary Fig. 8

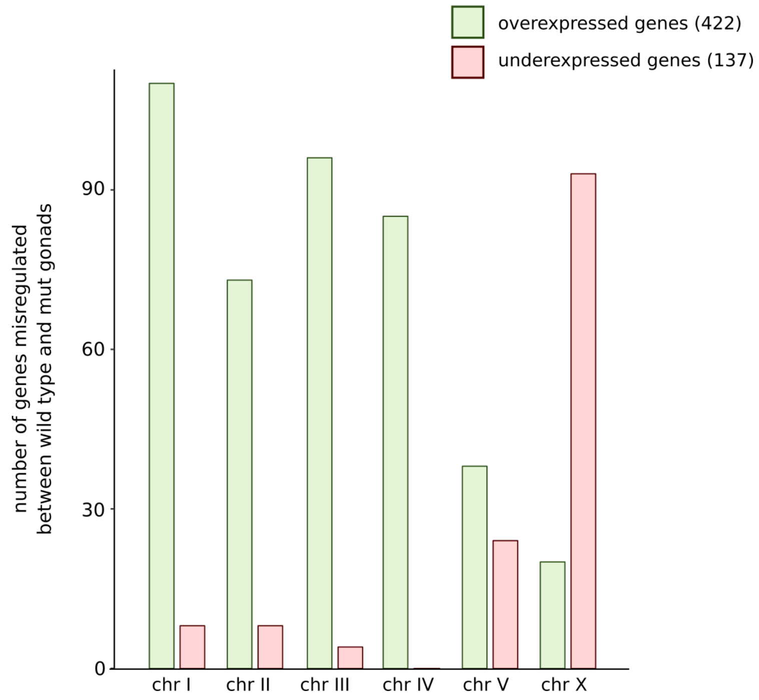

Supplementary Fig. 8. Number of genes misregulated in H3K27M mutant worms per chromosome. Number of overexpressed genes are represented by green bars, and number of underexpressed genes by red bars.

Supplementary Fig. 9

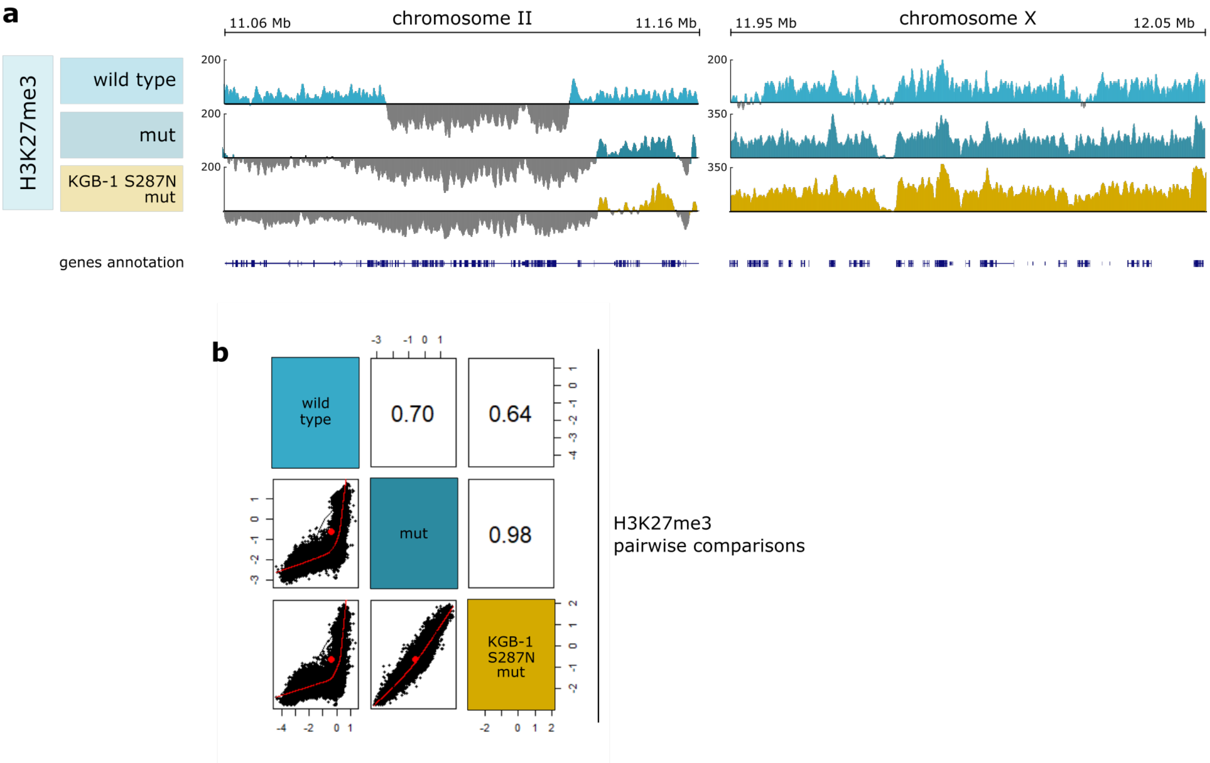

Supplementary Fig. 9. H3K27me3 distribution in H3.3K27M mutant worms is not affected by the JNK/KGB-1 S287N suppressor mutation (**a**) Genome browser view of 100 kb on chromosome II and X showing H3K27me3 distribution in wild type (light blue), H3.3K7M mutant (mut, dark blue) and mut; KGB-1 S287N (yellow) worms. (**b**) Pairwise genome-wide comparisons between H3K27me3 levels in wild type, mut and mut; KGB-1 S287N worms, in 1kb bins. Panels show scatter plots and Pearson correlations for each sample pair. The wild type and mut data are reproduced from Fig. 3a.

Supplementary Fig. 10

a

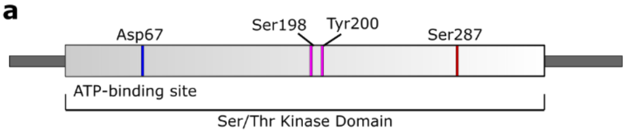

b

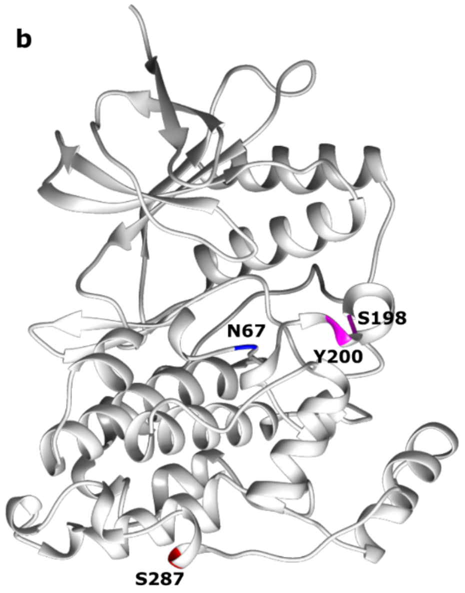

c

phosphosites prediction

#	x	Context	Score	Kinase	Answer
287	S	ARAFSEIVP	0.957	unsp*	YES
287	S	ARAFSEIVP	0.632	PKA	YES
287	S	ARAFSEIVP	0.573	cdc2	YES
287	S	ARAFSEIVP	0.548	PKG	YES

*unsp - unspecified kinase

Supplementary Fig. 10. The suppressor mutation in *kgb-1*/JNK may affect a regulatory site. (**a**) The identified KGB-1 S287N mutation differs from S198 and T200 that are required for kinase activity, and the annotated ATP-binding site N67 of the kinase domain. (**b**) The S287 residue (marked in red) is likely exposed to the surface of KGB-1. The KGB-1 structure was modeled with SWISS-MODEL using human JNK1 structure (PDB = 3VUG) as scaffold. S198 and T200 (required for kinase activity) are in orange and the annotated ATP-binding site N67 in blue. (**c**) Prediction of S287 as a highly likely phosphorylation target. Scores were obtained using NetPhos 3.1.

Supplementary Fig. 11

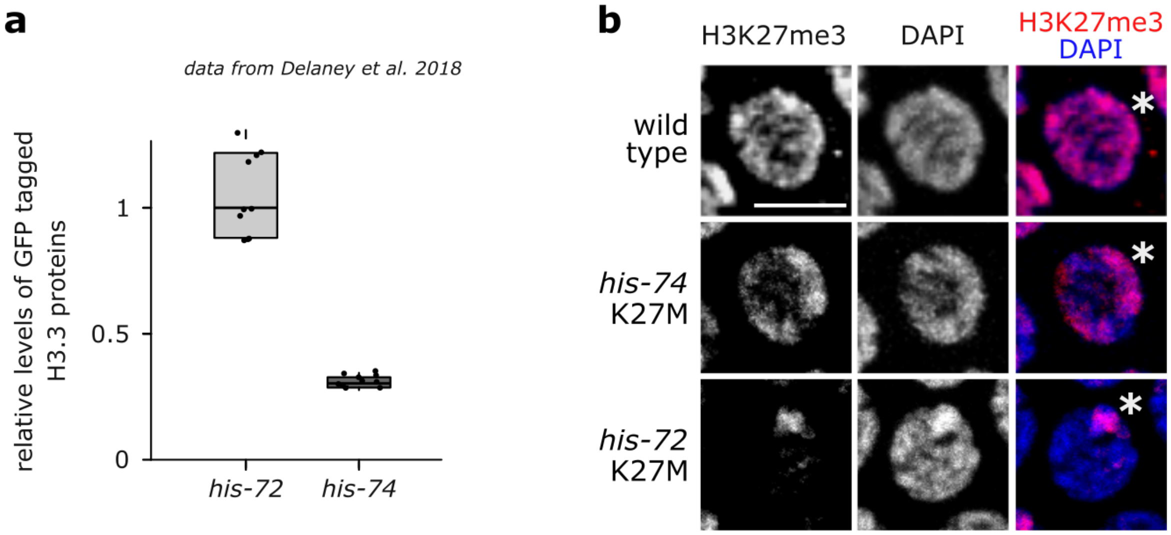

Supplementary Fig. 11. The H3.3 genes *his-72* and *his-74* have different expression levels and induce different degrees of H3K27me3 redistribution upon acquisition of the K27M mutation. (**a**) Relative levels of *his-72* and *his-74* expression in nuclei of germ cells carrying endogenously GFP-tagged copies of both H3.3 proteins (data from Delaney et al. 2018). Median levels of *his-72*::GFP signal were set to 1. Source data are provided as a Source Data Supplementary Fig. 11 file. (**b**) H3K27me3 staining in pachytene germline cells of wild type, HIS-72 K27M and HIS-74 K27M worms. Chromosome X is marked by an asterisks in the merged images. Scale bar represents 5 µm.

Supplementary Table 1. Overview of *C. elegans* strains used in this study.

**Strain**	**Genotype**	**Source**	**Comments**	**# alleles**
N2	wild type	CGC	wild type strain	
FAS58	*his-72(uge40[K27M]) III*	This study	H3.3K27M mutant	7
FAS100	*his-72(uge67[A87S A88V I89A G90M]) III*	This study	H3-like	1
FAS104	*his-72(uge68[K27M A87S A88V I89A G90M]) III*	This study	H3-like containing K27M mutant	1
FAS107	*his-72(uge69[OLLAS::his-72]) III*	This study	OLLAS-tagged H3.3	1
FAS119	*his-72(uge79[OLLAS::his-72 K27M]) III*	This study	OLLAS-tagged H3.3 K27M mutant	1
FAS138	*his-72(uge89[OLLAS::his-72 K27M A87S A88V I89A G90M]) III*	This study	OLLAS-tagged H3-like containing K27M mutant	2
FAS129	*his-72(uge40[K27M]) III, kgb-1(uge83[S287N]) IV*	This study	Suppressor EMS allele of *kgb-1*	1
FAS130	*his-72(uge40[K27M]) III, kgb-1(uge84[S287N]) IV*	This study	CRISPR allele of *kgb-1*	1
FAS146	*his-74(uge97[OLLAS::his-74 K27M]) V*	This study	OLLAS-tagged H3.3 (*his-74*) K27M mutant	1

Supplementary Table 2. Reagents used for allele generation by CRISPR/Cas9.

Mutation	sgRNA plasmids	sgRNA target sequences (PAM sites in bold)	Repair template	PCR primer 1 for edit detection	PCR primer 2 for edit detection
his-72[K27M] (uge40, uge68, uge79, uge89)	pFS115	GTTGGAGCCGATTT GCGGG**CGG**	oFSa0121	TCGCAATTTCCT ACACCTCC[1]	ACGAGACGTTGG AAAGGAAG[1]
his-72[A87S A88V I89A G90M] (uge67, uge68, uge89)	pKD0040 pKD0041	GCTTCCAGTCGGCT GCCAT**CGG** AGCTCCGATGGCA GCCGAC**TGG**	oKD0271	GACTACTGAAGC ACACGAAA[2]	CGACCGCAACGC GTAGGGC[2]
kgb-1[S287N] (uge84)	pKD0055	ATGCTCGTGCCTGA TGACG**TGG**	oKD0356	ATCCTGTATAGC CTGACTCACG[2]	TGGACTTCCATC TGCGCTC[2]
OLLAS::his-72 (uge69, uge79, uge89)	pKD066	GCTTAAGCACGTTC TCCG**CGG**	oKD0323	AGAACGTGCTAT GGGAGG	GTTTCGTGTGCT TCAGTAGTC
his-74[K27M] (uge97)	pFSa0122 pFSa0123	ACGAAGGCGGCTC GCAAGT**CGG** TTGACACTTCCGGT GACAA**TGG**	oFSa0143	GCTACCGTAACC CTCTTCAATC[3]	TGAAGAGCTCCG ATAGCAGC
OLLAS::his-74 (uge97)	pKD0106 pKD0107	GATCTAGCTGCGCT CTCCA**CGG** TTCGGGCAGTAATA AAACA**GGG**	oKD0407	GCTTTAATTTGG CCGTGTCGAG	GGATGAGAAATG TGCAGGTGTG

**Repair template sequences**	
oFSa0121	CCGGAGGAAAGGCTCCAAGAAAGCAGTTGGCCACCAAGGCAGCCCGCAT GTCGGCGCCAACCACCGGAGGAGTCAAGAAGCCACATCGTTACCGTC
oKD0271	GATTTTAATTAATTTTTTTAAATTTCAAATTCAAACTACAAACCTGCAGA GCCATGACAGCGGACGACTGGAAGCGGAGATCAGTCTTGAAGTCCTGGG CAATCTCACG
oKD0356	GGGAACACCAGATGATCATTTCATTAGTCAATTGGGTCAGTCAGCTGCTA TGTATGTTCGATCGTTGCCGCGTCATCAGGCACGCGCATTCAATGAAATC GTTCCTGATACCAATTTTCTGCCGGAGACTGAAAATCCAAGGGTCC
oKD0323	GTGCTTCGAGAATTGGTGATGGAGCTTACTTTCCCATGAGACGTGGTCCG AGCTCGTTGGCGAATCCGGAGGATCCTCCCATAGCACGTTCTCCGCGAAT GCGTCTGGC
oFSa0143	GAAAAGCTCTGGCGACGAAGGCGGCTCGCATGTCCGCAATTGTCACCGG AAGTGTCAAAAAAGTGCATC
oKD0407	GCCAGACGCATTCGTGGAGAGCGCAGCATGGGAGGATCCTCCGGATTCG CCAACGAGCTCGGACCACGTCTCATGGGAAAGTAGATCcATGTTTTATTA CTGCCCG

[1] Same size PCR product in wild type and *uge40*, but *uge40* contains a *Sfo*I restriction site.
[2] Same size PCR product in wild type and *uge29*, but *uge29* contains a *Pst*I restriction site.
[3] Same size PCR product in wild type and *uge93*, but *uge93* contains a *Mfe*I restriction site.
