# Supplementary material for: H3.3K27M-induced chromatin changes drive ectopic replication through misregulation of the JNK pathway in C. elegans
Source: Nat Commun. 2019 Jun 7;10:2529. doi: 10.1038/s41467-019-10404-9 (PMC6555832; doi:10.1038/s41467-019-10404-9)
Supplement: Supplementary file 3 — Description of Additional Supplementary Files [file 41467_2019_10404_MOESM3_ESM.pdf]

Description of Additional Supplementary Files

File Name: Supplementary Data 1
Description: Gene expression profiles of wild type and H3.3K27M mutant germlines.
