# Supplementary material for: H3.3K27M-induced chromatin changes drive ectopic replication through misregulation of the JNK pathway in C. elegans
Source: Nat Commun. 2019 Jun 7;10:2529. doi: 10.1038/s41467-019-10404-9 (PMC6555832; doi:10.1038/s41467-019-10404-9)
Supplement: Supplementary file 5 — Reporting Summary [file 41467_2019_10404_MOESM5_ESM.pdf]

natureresearch

Corresponding author(s): Florian A Steiner

Last updated by author(s): Apr 24, 2019

Reporting Summary

Nature Research wishes to improve the reproducibility of the work that we publish. This form provides structure for consistency and transparency in reporting. For further information on Nature Research policies, see Authors & Referees and the Editorial Policy Checklist.

Statistics

For all statistical analyses, confirm that the following items are present in the figure legend, table legend, main text, or Methods section.

n/a	Confirmed	
☐	☒	The exact sample size (*n*) for each experimental group/condition, given as a discrete number and unit of measurement
☐	☒	A statement on whether measurements were taken from distinct samples or whether the same sample was measured repeatedly
☐	☒	The statistical test(s) used AND whether they are one- or two-sided<br>*Only common tests should be described solely by name; describe more complex techniques in the Methods section.*
☐	☒	A description of all covariates tested
☐	☒	A description of any assumptions or corrections, such as tests of normality and adjustment for multiple comparisons
☐	☒	A full description of the statistical parameters including central tendency (e.g. means) or other basic estimates (e.g. regression coefficient) AND variation (e.g. standard deviation) or associated estimates of uncertainty (e.g. confidence intervals)
☐	☒	For null hypothesis testing, the test statistic (e.g. *F*, *t*, *r*) with confidence intervals, effect sizes, degrees of freedom and *P* value noted<br>*Give P values as exact values whenever suitable.*
☒	☐	For Bayesian analysis, information on the choice of priors and Markov chain Monte Carlo settings
☒	☐	For hierarchical and complex designs, identification of the appropriate level for tests and full reporting of outcomes
☐	☒	Estimates of effect sizes (e.g. Cohen's *d*, Pearson's *r*), indicating how they were calculated

Our web collection on statistics for biologists contains articles on many of the points above.

Software and code

Policy information about availability of computer code

Data collection	All genomic data was collected using Illumina HiSeq 2500 machines at the Genomics Platform of the iGE3 at the University of Geneva. Images were obtained using a Leica DM5000 B fluorescent microscope or a Leica SP8 confocal microscope.
Data analysis	For ChIP-seq data, raw reads of 50 bases were generated using Illumina HiSeq 2500. The raw sequencing data was aligned to the C. elegans reference genome (WBcel215) with Novoalign (default parameters), producing SAM files. Reads were converted to JSON files and then to BEDGraphs with UNIX and R scripts. Genome browser images were obtained using the Sushi Bioconductor package. Heatmaps and hierarchical clustering were performed with Morpheus software (https://software.broadinstitute.org/morpheus). Function approximation was performed with Solver, a Microsoft Excel add-in program.<br><br>For RNA-seq data, raw reads of 50 bases were generated using Illumina HiSeq 2500. The reads were mapped with the TopHat v2.0.13 (default parameters) software to the C. elegans reference genome (WBcel235). Read counts representing the total number of reads aligning to each genomic feature were produced from aligned reads by the Python software htseq-count (–mode = union, with HTSeq v.0.6.1) with the reference .gtf file. The normalization and differential expression analysis between samples was performed with the R/ Bioconductor package edgeR v.3.10.5 for the genes annotated in the reference genome.<br><br>Fluorescence images were processed using Fiji (Shindelin et al. 2012).

For manuscripts utilizing custom algorithms or software that are central to the research but not yet described in published literature, software must be made available to editors/reviewers. We strongly encourage code deposition in a community repository (e.g. GitHub). See the Nature Research guidelines for submitting code & software for further information.

Data

Policy information about availability of data

All manuscripts must include a data availability statement. This statement should provide the following information, where applicable:

- Accession codes, unique identifiers, or web links for publicly available datasets
- A list of figures that have associated raw data
- A description of any restrictions on data availability

Sequencing data has been deposited at the Gene Expression Omnibus (GEO) under accession number GSE117533. Raw and processed data deposited at the GEO were used in Figures 3 and 4, and Supplementary Figures 2, 4, 5, 6 , 8 and 9. The data are available for the reviewers (see below for access), but restricted for the public until the manuscript is published. For reviewer access, see below.

Field-specific reporting

Please select the one below that is the best fit for your research. If you are not sure, read the appropriate sections before making your selection.

☒ Life sciences ☐ Behavioural & social sciences ☐ Ecological, evolutionary & environmental sciences

For a reference copy of the document with all sections, see nature.com/documents/nr-reporting-summary-flat.pdf

Life sciences study design

All studies must disclose on these points even when the disclosure is negative.

Sample size	For the sterility assessment, in each repetition we examined 100 individual worms, which was sufficient to account for the phenotype variability. For ChIP-seq, each chromatin IP was performed on nuclei extracted from about 5'000'0000 worms. For RNA-seq, each replicate consisted of around 30 hand-dissected adult gonads. For cell line experiments, each replicate was based on measurements from 3 wells in 96-well plates, which reproduces the conditions performed in the original assay (Hashizume et al. 2014)
Data exclusions	For the sterility phenotype counts, we excluded worms that died before the end of the experiment, as the cause for an eventual absence of progeny could not be determined in this case.
Replication	Each ChIP-seq and RNA-seq analysis was performed at least in duplicate, and the results were always consistent. Sterility counts and phenotype assessment were performed at least in triplicate, and results, even in cases of phenotypes with larger variability, were consistent.
Randomization	Worms used for each biological replicate belonged to the same generation, and samples and controls were assessed in parallel. For replicates, we were using worms separated by at least one generation. All the worms within the analysis were subjected to the same conditions, unless otherwise indicated.
Blinding	The experimentators were not blinded to the experimental groups, as the phenotypes were quantifyable (e.g. presence or absence of progeny, staining intensity) and were not scored according to subjective measures.

Reporting for specific materials, systems and methods

We require information from authors about some types of materials, experimental systems and methods used in many studies. Here, indicate whether each material, system or method listed is relevant to your study. If you are not sure if a list item applies to your research, read the appropriate section before selecting a response.

Materials & experimental systems

n/a	Involved in the study	
☐	☒	Antibodies
☐	☒	Eukaryotic cell lines
☒	☐	Palaeontology
☐	☒	Animals and other organisms
☒	☐	Human research participants
☒	☐	Clinical data

Methods

n/a	Involved in the study	
☐	☒	ChIP-seq
☒	☐	Flow cytometry
☒	☐	MRI-based neuroimaging

Antibodies

Antibodies used	anti-H3K4me3 antibody (Abcam ab8580, lot:GR3175719-3)<br>anti-H3K27me2me3 antibody (Active Motif 39535, lot:30617013)<br>anti-H3K36me3 antibody (Abcam 9050, lot:GR3198867-1)<br>anti-OLLAS antibody (Novus Biologicals NBP1-06713B, lot:F-7)<br>anti-H3 phosphoS10 antibody (Abcam ab5176, lot:GR264582-1)

anti-POLD2 antibody (Thermo Fisher Scientific PA5-55401, lot:A106098)
anti-RAD-51 antibody (Novus Biologicals 29480002)
anti-BrdU antibody (BD535 Pharmingen 555627, lot:7033666)
anti-histone H3, abcam ab1791, lot:GR210026-1)

Validation

anti-H3K4me3 - used by us for IF, validated for that purpose in C. elegans by the manufacturer. Staining pattern in wildtype animals consistent with previously published results for the same modification.

anti-H3K27me3 - used by us for IF, WB, ChIP-seq, validated for all purposes by the manufacturer, validated for C. elegans by Gaydos et al. 2012. Staining and genome-wide incorporation patterns in wildtype animals consistent with previously published results for the same modification.

aanti-H3K36me3 - used by us for IF ChIP-seq, validated for those purposes and for C. elegans by the manufacturer. Genome-wide incorporation patterns in wild type animals consistent with previously published results for the same modification (Gaydos et al. 2012).

anti-OLLAS - used by us for IF, WB, ChIP-seq, validated for all purposes by the manufacturer, validated for C. elegans by Paix et al. 2017. Validated by absence of signal in worms not containing epitope.

anti-H3 phosphoS10 - used by us for IF and validated for that purpose by the manufacturer, not validated for C. elegans, but in wild type animals reproduced staining for the same mark in Jer-Yuan Hsu et al. 2000.

anti-POLD2 - used by us for IF and validated for that purpose by the manufacturer, not previously validated for C. elegans. Staining pattern in wildtype animals as expected from other replication markers.

anti-RAD51 - used by us for IF and validated for that purpose and for C. elegans by Waters et al 2012. Staining pattern in wildtype animals consistent with previously published results for the same protein.

anti-histone H3 - used by us for IF and WB, validated for that purpose and for C. elegans by the manufacturer.

anti-BrdU - used by us for IF and validated for that purpose and for C. elegans by Crittenden & Kimble, 2008. Staining pattern in wildtype animals as expected from other replication markers.

Eukaryotic cell lines

Policy information about cell lines

Cell line source(s)	SF8628 cell line - purchased from Merck (SCC127)<br>SF9402 and SF9427 - provided by Prof. Rintaro Hashizume (Northwestern University)
Authentication	Cell lines were not identicated.
Mycoplasma contamination	Cell lines were not tested on mycoplasma presence.
Commonly misidentified lines (See ICLAC register)	No commonly misidentified cell lines were used in this study.

Animals and other organisms

Policy information about studies involving animals; ARRIVE guidelines recommended for reporting animal research

Laboratory animals	No laboratory animals were used in this study.
Wild animals	No wild animals were used in this study.
Field-collected samples	Study did not involve samples collected in the field.
Ethics oversight	No ethical approval or guidance was required, as the study used nematode C. elegans as a model system.

Note that full information on the approval of the study protocol must also be provided in the manuscript.

ChIP-seq

Data deposition

☒ Confirm that both raw and final processed data have been deposited in a public database such as GEO.

☒ Confirm that you have deposited or provided access to graph files (e.g. BED files) for the called peaks.

Data access links
May remain private before publication.

To review GEO accession GSE117533:
Go to https://www.ncbi.nlm.nih.gov/geo/query/acc.cgi?acc=GSE117533
Enter token qrwjaicipxqlrsd into the box

Files in database submission

N2_H3K27me3_100bp_delta.bed
N2_H3K27me3_1kb_ratio.bed
N2_H3K27me3_1kb_logratio.bed
N2_H3K27me3_10kb_logratio.bed
N2_H3K36me3_100bp_delta.bed
N2_H3K36me3_1kb_logratio.bed
FAS58_H3K27me3_100bp_delta.bed
FAS58_H3K27me3_1kb_ratio.bed
FAS58_H3K27me3_1kb_logratio.bed
FAS58_H3K27me3_10kb_logratio.bed
FAS58_H3K36me3_100bp_delta.bed
FAS58_H3K36me3_1kb_logratio.bed
FAS100_H3K27me3_100bp_delta.bed
FAS100_H3K27me3_1kb_logratio.bed
FAS100_H3K27me3_10kb_logratio.bed
FAS104_H3K27me3_100bp_delta.bed
FAS104_H3K27me3_1kb_logratio.bed
FAS104_H3K27me3_10kb_logratio.bed
FAS107_H3.3::OLLAS_100bp_delta.bed
FAS107_H3.3::OLLAS_1kb_lograto.bed
FAS107_H3.3::OLLAS_10kb_lograto.bed
FAS119_H3.3K27M::OLLAS_100bp_delta.bed
FAS119_H3.3K27M::OLLAS_1kb_logratio.bed
FAS119_H3.3K27M::OLLAS_10kb_logratio.bed
FAS138_H3-likeK27M::OLLAS_100bp_delta.bed
FAS138_H3-likeK27M::OLLAS_1kb_logratio.bed
FAS138_H3-likeK27M::OLLAS_10kb_logratio.bed
N2_gonads_1kb_just_reads.bed
FAS58_endo_1kb_just_reads.bed
N0_i_me3_L1_R1_001_Tx7RjhFo9Das.fastq
N0_i_me3_L1_R2_001_oF5daS6kYaHq.fastq
N0_b_me3_L1_R1_001_akHe5aLqp6la.fastq
N0_b_me3_L1_R2_001_djzhG9anI1kw.fastq
N2_i_27d_L2_R1_001_as5dHJse9aDF.fastq
N2_i_27d_L2_R2_001_awq6gDFt7hAz.fastq
N2_b_27d_L2_R1_001_a5RfTgashy7Ua.fastq
N2_b_27d_L2_R2_001_sdr6TFgyH0Ai.fastq
N2_i_27_L1_R1_001_8Hathd5ahYD.fastq
N2_i_27_L1_R2_001_trg3sJKyr7wd.fastq
N2_b_27_L1_R1_001_2dGFthFa7wJh.fastq
N2_b_27_L1_R2_001_p9Las7hGfTy2.fastq
B6_L1_R1_001_NvPLGK1e8zuB.fastq
B6_L1_R2_001_npV0GhVS0tBk.fastq
B9_L1_R1_001_rXS5WQUbAIes.fastq
B9_L1_R2_001_QjdvrqCvOjEf.fastq
A6_L1_R1_001_1qF1lg2jlpVO.fastq
A6_L1_R2_001_eJcWWEku8pp2.fastq
A9_L1_R1_001_qyaslwAAERsk.fastq
A9_L1_R2_001_vh0pI251nK4V.fastq
J6_L1_R1_001_3wgcpGRS6Yvf.fastq
J6_L1_R2_001_S2uwS1UCBqFl.fastq
J9_L1_R1_001_sb8ka2dBfHaz.fastq
J9_L1_R2_001_R7DVcR4KY2dY.fastq
F585_i_me3_L2_R1_001_md6KAIv6385v.fastq
F585_i_me3_L2_R2_001_uAy5ZAzVpemX.fastq
F585_b_me3_L2_R1_001_EYWoVDlR3jJZ.fastq
F585_b_me3_L2_R2_001_S3w2te82vf6i.fastq
58_i_27d_L5_R1_001_BqHbpUOWcR46.fastq
58_i_27d_L5_R2_001_8uRRIM8Y5bMB.fastq
58_b_27d_L5_R1_001_lljOi7fQxhin.fastq
58_b_27d_L5_R2_001_UlZauuFpQwtt.fastq
58_i_27_L5_R1_001_hOhbyAaLv5RE.fastq
58_i_27_L5_R2_001_yntbGWnhwPT3.fastq
58_b_27_L5_R1_001_IFngFFLlxYzI.fastq
58_b_27_L5_R2_001_Cof81bzqYRqR.fastq
L6_L1_R1_001_MOtSwAsRpx0P.fastq
L6_L1_R2_001_fzFFP4IkpYgg.fastq
L9_L1_R1_001_1cPXHvsTEY2d.fastq
L9_L1_R2_001_V7XzoJRHxQ5s.fastq
D6_L2_R1_001_ABDfJZANme8L.fastq

D6_L2_R2_001_qtNHzJ21kZbE.fastq
D9_L2_R1_001_bp0ZVyD2CqiU.fastq
D9_L2_R2_001_Vdy5Of8LQQ7S.fastq
K3_L1_R1_001_wGCmAqNN3tvW.fastq
K3_L1_R2_001_6LYdE37rSidd.fastq
K4_L1_R1_001_J6woMrtN4ORG.fastq
K4_L1_R2_001_cqv3lqLwWth0.fastq
G6_L1_R1_001_kleuPONxMajB.fastq
G6_L1_R2_001_jEZumjFn6fG7.fastq
G9_L1_R1_001_Tv71FYjwYuIx.fastq
G9_L1_R2_001_5dIUD00kxC7n.fastq
Z3_L4_R1_001_jjUfgRP0IgCm.fastq
Z3_L4_R2_001_onkbzRLF4wuX.fastq
Z4_L4_R1_001_1NIJ7IZsKkGR.fastq
Z4_L4_R2_001_w8l95mzgra5B.fastq
H6_L1_R1_001_AuVU0tGB2MTG.fastq
H6_L1_R2_001_abVUodUTdgBD.fastq
H9_L1_R1_001_SMOFYBGIgkUX.fastq
H9_L1_R2_001_MtKXAhrsYGW8.fastq
PS27_L4_R1_001_iJKC2rGnM5Cc.fastq
PS27_L4_R2_001_lAl5TGDDVUVw.fastq
PS28_L4_R1_001_I2vGEvqc27tu.fastq
PS28_L4_R2_001_iSNMptITYzzW.fastq
PS29_L4_R1_001_Al13OzBrJtbk.fastq
PS29_L4_R2_001_eJrDW66sk78C.fastq
PS30_L4_R1_001_D9NrrKLBZJPg.fastq
PS30_L4_R2_001_XN2hTwvu8upT.fastq
A12_L1_R1_001_GrxOi1ed63ah.fastq
A12_L1_R2_001_0wEE4gJ0K0MP.fastq
A13_L1_R1_001_8SBeqfnOenaM.fastq
A13_L1_R2_001_oIcIPd3nqNzl.fastq
PS15_L3_R1_001_9GWbKclaETP9.fastq
PS15_L3_R2_001_sS1Z5ZNkvquK.fastq
PS16_L3_R1_001_EtpQYVxHmrsC.fastq
PS16_L3_R2_001_3jkFzrHAREFi.fastq
PS17_L3_R1_001_uBU8xsHVCsq3.fastq
PS17_L3_R2_001_HzFYghS1uYPq.fastq
PS18_L3_R1_001_3DD93ajGteJ1.fastq
PS18_L3_R2_001_eMJYJGQPG1P8.fastq
A16_L1_R1_001_mudLlxxmbXw0.fastq
A16_L1_R2_001_QXgm5E5krxzN.fastq
A17_L1_R1_001_OUxD41YNN9SA.fastq
A17_L1_R2_001_u4QCxQ3zfcwj.fastq
Z7_L4_R1_001_86IqoKDKwrlW.fastq
Z7_L4_R2_001_vJJhvC9ZJeTN.fastq
Z8_L4_R1_001_xk39kaySWYtp.fastq
Z8_L4_R2_001_fFQhA5oTD5h8.fastq
1A_L1_R1_001_WBfN2YKfw6Tg.fastq
1A_L1_R2_001_nFJeOYUg0LEi.fastq
1B_L1_R1_001_q6jEaSgVT9GQ.fastq
1B_L1_R2_001_W1hUMW0JKvr6.fastq
1C_L1_R1_001_OC1p555GoiU9.fastq
1C_L1_R2_001_P85b4DANl04j.fastq
1D_L1_R1_001_7sZBqtXmqayq.fastq
1D_L1_R2_001_7QUnBsCCkZwB.fastq
1E_L1_R1_001_jtisCF3pgxzV.fastq
1E_L1_R2_001_8xb7HnuNp9k8.fastq
1F_L1_R1_001_k4jwPZ1Ul1wD.fastq
1F_L1_R2_001_8APeDxSzdfoX.fastq
1G_L1_R1_001_aosjTYiCuBMU.fastq
1G_L1_R2_001_pxkya38IHkgD.fastq
1H_L1_R1_001_LaqnLzTEQCnM.fastq
1H_L1_R2_001_I6pmldAAgTeB.fastq
N2_2_L8_R1_001_cG0ZVwlKUnjG.fastq
endo_2_L8_R1_001_OyYYnKlyE0gH.fastq
C6_L2_R1_001_JdMDD4yjap2T.fastq
C6_L2_R2_001_z7O8dNUpDCCq.fastq
K6_L1_R1_001_tayMjNPPZZPc.fastq
K6_L1_R2_001_UCm6dvHsJeQX.fastq
C9_L2_R1_001_unC6BJQBTdo2.fastq
C9_L2_R2_001_ullfet8kM4ph.fastq
K9_L1_R1_001_JHQ6KwnU44t4.fastq
K9_L1_R2_001_4iSFT6wVqDHA.fastq
E6_L2_R1_001_3rP6MxskSWJ6.fastq
E6_L2_R2_001_jckchdD7I8bt.fastq

M6_L1_R1_001_C1KKhzEf47WU.fastq
M6_L1_R2_001_Vh8pEy0J8FGh.fastq
E9_L2_R1_001_r498aHCskslq.fastq
E9_L2_R2_001_X55zrRhMD94B.fastq
M9_L1_R1_001_E2h92wfp5IT8.fastq
M9_L1_R2_001_ekpx47SZoeQl.fastq

Genome browser session
(e.g. UCSC)

https://genome.ucsc.edu/cgi-bin/hgTracks?
hgS_doOtherUser=submit&hgS_otherUserName=kdelaney&hgS_otherUserSessionName=Delaney_et_al

Methodology

Replicates

All of the final files are build on several biological replicates. For each experiment we performed:
N2_H3K27me3_ChIP - 6 biological replicates
N2_H3K36me3_ChIP - 2 biological replicates
FAS58_H3K27me3_ChIP - 5 biological replicates
FAS58_H3K36me3_ChIP - 2 biological replicates
FAS100_H3K27me3_ChIP - 3 biological replicates
FAS104_H3K27me3_ChIP - 3 biological replicates
N2_H3.3::OLLAS_ChIP - 3 biological replicates
FAS119_H3.3K27M::OLLAS_ChIP - 2 biological replicates
FAS138_H3-likeK27M::OLLAS_ChIP - 4 biological replicates

Sequencing depth

In all ChIP-seq experiments reads are 50 bp and paired-end.
N2_H3K27me3_input - 177 630 541 total reads, 137 533 233 uniquely mapped reads (77.4%)
N2_H3K27me3_IP - 174 228 809 total reads, 129 388 719 uniquely mapped reads (74.3%)
N2_H3K36me3_input - 22 775 747 total reads, 18 422 073 uniquely mapped reads (77.5%)
N2_H3K36me3_IP - 24 333 508 total reads, 18 680 290 uniquely mapped reads (76.8%)
FAS58_H3K27me3_input - 156 762 094 total reads, 111 912 049 uniquely mapped reads (71.4%)
FAS58_H3K27me3_IP - 143 817 278 total reads, 106 161 433 uniquely mapped reads (73.8%)
FAS58_H3K36me3_input - 14 285 999 total reads, 11 412 271 uniquely mapped reads (79.9%)
FAS58_H3K37me3_IP - 24 685 561 total reads, 18 667 111 uniquely mapped reads (75.6%)
FAS100_H3K27me3_input- 76 387 554 total reads, 51 461 701 uniquely mapped reads (67.4%)
FAS100_H3K27me3_IP - 40 708 896 total reads, 32 717 068 uniquely mapped reads (80.4%)
FAS104_H3K27me3_input - 72 168 248 total reads, 52 966 138 uniquely mapped reads (73.4%)
FAS104_H3K27me3_IP - 86 263 523 total reads, 63 874 164 uniquely mapped reads (74%)
N2_H3.3::OLLAS_input- 124 937 534 total reads, 81 255 405 uniquely mapped reads (65%)
N2_H3.3::OLLAS_IP- 144 240 606 total reads, 107 270 141 uniquely mapped reads (74.4%)
FAS119_H3.3K27M::OLLAS_input - 39 660 897 total reads, 31 533 649 uniquely mapped reads (79.5%)
FAS119_H3.3K27M::OLLAS_IP - 74 934 893 total reads, 58 906 190 uniquely mapped reads (78.6%)
FAS138_H3-likeK27M::OLLAS_input- 147 494 048 total reads, 111 758 169 uniquely mapped reads (75.8%)
FAS138_H3-likeK27M::OLLAS_IP - 177 721 679 total reads, 135 833 007 uniquely mapped reads (76.4%)

Antibodies

anti-H3K27me3 - validated for ChIP by the manufacturer, validated for C. elegans by Gaydos et al. 2012
anti-H3K36me3 - validated for ChIP and C. elegans the manufacturer
anti-OLLAS - validated for ChIP purpose by the manufacturer, validated for C. elegans by Paix et al. 2017

Peak calling parameters

We did not perform peak calling in this study.

Data quality

To ensure the quality of ChIP experiment we assesed the lenght of our reads and rejected the ones that do not fit in the expected range in the process of alignment. We also correlated repeats with each other to ensure we are merging robust, consistent repeats.

Software

For ChIP-seq data, raw reads of 50 bases were generated using Illumina HiSeq 2500. The raw sequencing data was aligned to the C. elegans reference genome (WBcel215) with Novoalign (default parameters), producing SAM files. Reads were converted to JSON files and then to BEDGraphs with UNIX and R scripts. All files were normalized to the same number of reads. For each ChIP experiment, IP files were normalized using the input file with the corresponding bin size. Biological replicates of ChIP-seq experiments that passed the quality control were merged on the level of aligned reads (SAM files), and the downstream pipeline was performed again using the merged file and smoothed to obtain the final normalized bedgraph for each experiment. Processed data files are bedgraphs containing normalized ChIP results. Normalization was performed by input subtraction (to avoid low number of read biases; marked in the file name by_delta) or by division of input followed by log2 transformation (marked in the file name by_logratio). Bin sizes used in the analysis were 100 bp (marked in the file name by _100bp), 1 kb (marked in the file name by _1kb) or 10 kb (marked in the file name by _10kb). The appropriate bin size was selected for each analysis. Genome browser images were obtained using the Sushi Bioconductor package. Heatmaps and hierarchical clustering were performed with Morpheus software (https://software.broadinstitute.org/morpheus). Function approximation was performed with Solver, a Microsoft Excel add-in program.
